# Anti-SARS-CoV-2 Antibody Response Among Spectators of Amir Cup 2020 With a History of Recovery From COVID-19 in Qatar: A Historic Cohort Study

**DOI:** 10.7759/cureus.54406

**Published:** 2024-02-18

**Authors:** Jazeera Saidarakath, Brijeshkumar Joravarsinh Gadhavi, Marwa Osman Awad, Muna Mehdar AlSaadi, Manshad Chovar Kattil, Ahmed Sameer Alnuaimi

**Affiliations:** 1 Clinical Pathology, Primary Health Care Corporation, Doha, QAT; 2 Family Medicine, Primary Health Care Corporation, Doha, QAT; 3 Laboratory, Primary Health Care Corporation, Doha, QAT; 4 Clinical Research, Primary Health Care Corporation, Doha, QAT

**Keywords:** severe acute respiratory syndrome coronavirus 2 (sars-cov-2), qatar, reinfection, antibody, immune response

## Abstract

Aim

The aim of the study is to describe the antibody response after COVID-19 infection and assess its effectiveness against reinfection.

Background

COVID-19 has recently emerged as a contagious infectious disease caused by Severe Acute Respiratory Syndrome Coronavirus 2 (SARS-CoV-2). This infection is followed by a humoral immune antibody response, which may remain in the blood for a number of weeks. Studies have shown that antibodies protect against reinfection for at least seven months. The current study is aimed at investigating the persistence of circulating SARS-CoV-2 antibodies after COVID-19 infection and its behavior over 18 months of follow-up period, in addition to assessing the risk of reinfection of COVID-19 in unvaccinated individuals.

Methodology

A longitudinal historical cohort study of 3378 COVID-19 recovered individuals in connection with the Amir Cup football tournament held in Qatar, in December 2020 was analyzed. The health records of study participants were followed for a maximum of 18 months after serology testing or until the first dose of COVID-19 vaccination to detect any evidence of recurrent infection.

Results

The study found a statistically significant association between recurrence risk and the duration of risk exposure since the first COVID-19 episode. Compared to those with the lowest risk of exposure to reinfection (shortest duration after first infection) those beyond 299 days of at-risk exposure since the first episode, have a 51-fold higher risk of developing recurrent COVID-19.

Conclusion

Immunity developed after primary infection with SARS-CoV-2 may protect against reinfection from subsequent exposure to the virus in seropositive individuals up to nine months post-infection.

## Introduction

COVID-19 infection is followed by humoral immune response through antibody production, starting as early as the third day after infection. In milder cases, antibody detection may take longer time. After Severe Acute Respiratory Syndrome Coronavirus 2 (SARS-CoV-2) infection, antibodies were detectable within one to two weeks following infection, with levels rising till 14 to 22 days and later decreasing, but titers were lesser in asymptomatic or clinically mild cases like in the study by Post et al. [1]. Detection of these antibodies, however, may not directly correlate with protective immunity. The longevity of these antibody responses is still controversial. SARS-CoV-2 IgG and IgM may last for seven weeks in blood [2].

During the pandemic declared by WHO, the validated serological assays of antibodies were crucial for contact tracing, identifying viral reservoir hosts, and for epidemiologic studies. Antibody assays as part of epidemiological studies helped to uncover the burden of disease, especially the asymptomatic infection, and to get better estimates of illness and death. In addition, validated serological tests were used in epidemiological studies to identify the extent of virus spread in households and communities, which could help guide control measures. The serological test is also needed for the evaluation of the results of vaccine trials and the development of therapeutic antibodies [3].

In relation to the production of antibodies in SARS-CoV-2 infections, the detection of IgM antibodies occurs from the fourth day of infection, increasing with time until reaching the 20th day (approximate peak) and reducing. The detection of IgG antibodies, on the other hand, starts from the 7th day peaks on the 25th day, and maintains high levels after four weeks of infection [4].

In the production of antibodies according to the severity of the disease, it is observed that three days after the onset of symptoms, the IgM titers gradually increase in patients with mild and severe forms over time [5]. In the work developed by Long et al. [6], it was mentioned that the IgG and IgM titers were high in the severe group compared to the non-severe group, with statistical difference in IgG two weeks after the onset of symptoms. Furthermore, the seronegative patients evaluated presented seroconversion of IgM or IgG 20 days after the onset of symptoms.

A large serologic survey in Qatar, in 2020 on 2102 individuals showed that IgG antibody concentrations potentially influence the risk of subsequent SARS-COV-2 reinfection [7]. Xiao et al. [8] found that in the time profile of detection of IgM and IgG after COVID-19 infection, IgM and IgG were positive three weeks after infection. IgG titer increased in the fourth week and IgM titer decreased. At seven weeks IgM was negative in a few patients and IgG was persisting in all. Detectable and continuous high levels of IgM indicated the acute phase of infection. Furthermore, IgM lasts more than a month indicating the prolonged virus replication in SARS-CoV-2 infected patients. IgG responded later than IgM and persisted high in indicating the humoral immune reaction to protect the body against the SARS-CoV-2 virus.

Kellam and Barclay [2] found that antibody response is more in severe cases and there is a paucity of information about the longevity of the antibody response to SARS-CoV-2, but it is known that antibodies to other human coronaviruses wane over time. Zhao et al. [9] reported a positive correlation between the severity of infection and antibody titer and noticed that there is no correlation between the rising titer of antibodies and RNA clearance.

In Qatar, 40% of the population constitutes the urban population and about 60% of the population consists of labor workers [10]. The current study included the population irrespective of the urban or labor category. Anti-SARS-CoV-2 antibody testing was done for the spectators of the Amir Cup 2020 who already had COVID-19 infection and recovered. Analysis was being done to verify the antibody response of spectators who did the blood test after receiving instructions to perform the antibody screening test. Blood samples were collected at different health centers all over the country for anti-SARS-CoV-2 antibodies and sent for testing. These serological tests, which detect antibodies against SARS-CoV-2, helped to detect previously COVID-19-infected individuals and to assess the extent of exposure of a population. It also helped to decide on the application, enforcement, or relaxation of containment measures. In SARS-CoV-2 infection, antibodies targeting both the spike and nucleocapsid proteins, which correlate with a strong neutralizing response, are formed as early as day nine onwards, suggesting seroconversion may lead to protection for at least a limited time [11]. SARS-CoV-2 antibody tests are not recommended for diagnosis of a current infection with COVID-19 [12].

Patients attain adaptive immune responses following SARS-CoV-2 infection, triggering antiviral humoral and cellular immune responses via B and T cell-mediated immunity. Seroconversion or a positive antibody test result obtained soon after the onset of infection was strongly associated with protection against reinfection [13]. Although it has been speculated that the development of antibodies may be associated with a decreased risk of reinfection, Hoang et al. [14] found that reinfection in patients with SARS-CoV-2 antibodies shows that seropositivity might be associated with limited protection against different viral strains. A comparative evolutionary study by Townsend et al. concluded that reinfection by SARS-CoV-2 under endemic conditions would likely occur between 3 months and 63 months after peak antibody response, with a median of 16 months [15]. Mehmet et al. published that humoral antibodies against SARS-CoV-2 were protective against COVID-19 reinfection, 0.8% of the patients had reinfection and the resultant reinfection was mostly seen in PCR-negative patients who were asymptomatic [16].

## Materials and methods

Aim of the study

The aim of the study is to describe the antibody response after COVID-19 infection and assess its effectiveness against reinfection. (I) To estimate the prevalence of anti-SARS-CoV-2N antibodies in COVID-19 recovered spectators of the Amir Cup 2020. The age, gender, and nationality stratified prevalence rate will be estimated; (II) to establish a correlation between the titer of antibodies and time duration from infection; (III) to describe the sero-reactivity status of anti-SARS-CoV inter N in the study population; and (IV) to estimate the COVID-19 re-infection rate in the studied population within 18 months after the initial antibody test.

Study design

This is a retrospective analysis of stored electronic data of recruited patients for whom COVID-19 antibody tests were done and analyzed. Data was collected from an electronic information system - Cerner. The date of the first COVID-19 polymerase chain reaction test and the date of declaration of cured status (first negative test result) were requested and correlated with the antibody titer. Demographic data like age and sex also were collected.

Study sample

The individuals for whom anti-SARS-COV-2 antibody testing was performed in Qatar in connection with the Amir Cup football tournament 2020 and the opening ceremony of Al Rayyan Stadium, Al Rayyan, Qatar, in December 2020.

Study method

During the COVID-19 pandemic, the Qatar surveillance system for COVID-19 was centrally managed by the Primary Health Care Corporation (PHCC). Any suspected case or its contacts would be tested by real-time reverse transcriptase-polymerase chain reaction (rtPCR) and its result stored in the Cerner (electronic health record) system. Anti-SARS-CoV-2 antibody serologic testing was done in Qatar in connection with the Amir Cup football final tournament (the opening ceremony of Al Rayyan Stadium held on December 18, 2020). The positive sero-reactive test result was considered evidence of immune response against COVID-19 (surrogate evidence for being protected against COVID-19) and considered a criterion for eligibility to access the stadium for that occasion. Testing was done for those individuals with a documented history of COVID-19 infection (positive rtPCR test result, which is the golden standard test) followed by recovery (negative rtPCR test result). Simple messaging service invitations were sent to a subset of the previously described population, who expressed interest in attending the tournament to perform the antibody test. An anti-SARS-CoV-2 order has been automatically placed on the electronic health record system (Cerner) for the Amir Cup 2020 screening. Samples collections were done at PHCC and the results were used to collate a list of eligible spectators. All the population values were used for statistical analysis and no sampling was practiced.

Laboratory methods and procedure

In PHCC Health centers, specimens were taken from each participant by certified laboratory technologists. The participant's median cubital vein on the forearm was used to draw blood into a yellow top gel tube using a vacutainer blood collection system. A 2-8-degree temperature-controlled transport container was used to transport specimens to the referral virology laboratory for testing. In the virology laboratory qualitative detection of antibodies against the SARS-CoV-2 antinucleocapsid (N) protein and semiquantitative detection of titer of antibodies against the SARS-CoV-2 spike protein receptor-binding domain (RBD) using electrochemiluminescence immunoassay under stringent quality-controlled procedure. Through a biphasic interphase system of the Cerner Homeless Management Information System software, test results were automatically transferred to each patient file and data was provided by the Computer Information Science department after approval of request by the PHCC research department.

Quality assurance

To ensure the accuracy of the data, a two-stage validation process was undertaken by the Business Health Intelligence department in PHCC (the data custodian). The codes used to extract data from Cerner were developed by a health information management specialist and reviewed by another one to ensure their accuracy in extracting the required dataset.

Data management

The following variables were extracted from Cerner: age, gender, and nationality. The date of the most recent COVID-19 rtPCR positive result and that of the following negative test result (all these dates should precede December 18, 2020). The date of the anti-SARS-COV-2 antibody test and its titer. The most recent positive test result occurred after December 18, 2020, within the following 18 months of follow-up. Vaccination status of the study population (the date of each vaccine dose during the 18-month follow-up period).

The previously listed variables were used to calculate the following intervals: Time interval in days from the last positive PCR test (first documented COVID-19 episode) until the serologic testing. Duration of risk exposure to reinfection (from the first positive rtPCR test result until the first vaccine dose or the total follow-up for unvaccinated individuals). The possible protective effect of vaccination that will confound the risk of infection was adjusted for by removing the vaccinated individual from the risk exposure status once vaccinated. The intervals were categorized using the quintiles method. The cut-off values for defining three-thirds (terciles) were calculated for the current population data.

Statistical analysis

The statistical analysis was executed using IBM SPSS Statistics for Windows, Version 28 (Released 2021; IBM Corp., Armonk, New York, United States). Frequency distributions were performed. The statistical significance of the association between two categorical variables was tested by the chi-square test of independence.

The antibody titer is a non-normally distributed quantitative variable. Its average is best described by median and the non-parametric test of significance are the best analytic statistical tools that apply. The difference in average between the two groups was tested for statistical significance by the Mann-Whitney test, while between more than two groups the Kruskal-Wallis test was applied.

A multiple logistic regression model was used to assess the risk of having a COVID-19 recurrence during 18 months of the follow-up period after serology testing during the unvaccinated period. The model allows for calculating the net and independent effect of each of a set of explanatory variables on the risk of having the outcome after adjusting for the possible confounding effect of the rest.

Ethical approval

The study was approved by the Institutional Review Board at the Primary Health Care Corporation, Qatar (PHCC/DCR/2022/09/051).

## Results

The results of this study were based on the analysis of health records of 3,378 individuals who recovered from a documented COVID-19 infection (based on positive results of the golden standard test rtPCR). The highest proportion of the study sample (58.3%) were young adults 20-39 years old. The study sample was based on attendants of a football match; therefore, more than three-quarters were male (77.4%). Qatari nationality represented only 8.9% of the sample (Table 1).

**Table 1:** Description of the study group

		N	%
1.	Age group (years)		
	10-19	197	5.8
	20-29	550	16.3
	30-39	1420	42.0
	40-49	811	24.0
	50+	400	11.8
	Total	3378	100.0
2.	Gender		
	Female	762	22.6
	Male	2616	77.4
	Total	3378	100.0
3.	Nationality		
	Expat	3076	91.1
	Qatari	302	8.9
	Total	3378	100.0

The study sample had to provide evidence of immune response against COVID-19 to approve their attendance at the mass gathering event in the football match. They voluntarily agreed to have a serological test for COVID-19. The prevalence of sero-reactivity in the current study was (3135/3378) 92.8%.

Table 2 explored the association between age, gender, nationality, and time interval since the last positive PCR test result with the detected COVID-19 antibody titer. All the tested explanatory variables had a statistically significant association with antibody titer. The median antibody titer was highest at 73.8 in older ages (50+ years) and lowest at 21.1 and 37.4 in those 20-29 and 30-39 years old, respectively. Males had a higher median titer (45.1) compared to females (35.9). Qataris had a lower average titer (28.4) compared to expats (44.4). Finally, the average antibody titer showed an obvious decline with time since the last episode of COVID-19 infection. Those with the highest duration (third tercile > 186 days since infection) had the lowest average antibody titer (31.1) compared to those with the most recent infection episode (first tercile < 112 days since infection) (Table 2).

**Table 2:** The median antibody titer by selected explanatory variables PCR: Polymerase chain reaction

		Antibody titer				
		Range	Median	Interquartile range	N	p
1.	Age group (years)					<0.001
	10-19	(0.1 to 150)	55.1	(18.1 to 140)	197	
	20-29	(0.1 to 150)	21.1	(5.7 to 61)	550	
	30-39	(0.1 to 150)	37.4	(10.35 to 102)	1420	
	40-49	(0.1 to 150)	54.6	(16.9 to 116)	811	
	50+	(0.1 to 150)	73.8	(22.3 to 133)	400	
	Total	(0.1 to 150)	42.5	(11.4 to 109)	3378	
2.	Gender					<0.001
	Female	(0.1 to 150)	35.9	(8.3 to 92.8)	762	
	Male	(0.1 to 150)	45.1	(12.5 to 113.5)	2616	
3.	Nationality					<0.001
	Expat	(0.1 to 150)	44.4	(11.8 to 110)	3076	
	Qatari	(0.1 to 150)	28.4	(6.5 to 90.5)	302	
4.	Time interval since last positive PCR test (days) - categories					<0.001
	First tercile (lowest) <= 112	(0.1 to 150)	55.0	(17.8 to 118)	1129	
	Second tercile (average) 113-186	(0.1 to 150)	42.3	(11.5 to 107)	1133	
	Third tercile (highest) > 186	(0.1 to 150)	31.1	(7.5 to 89.4)	1116	

The study group's health records were followed for a maximum of 18 months after serology testing or until the first dose of COVID-19 vaccination to detect any evidence of recurrent infection (positive rtPCR test result). The overall recurrence rate was 1.3%. The recurrence rate showed a statistically significant association with age group. The rate was highest (2%) in younger ages (10-19 and 20-29 years old) and lower in older ages. Gender showed no obvious or statistically significant association with the recurrence rate. Qatari nationality had an obviously higher recurrence rate (2%) compared to expats (1.2%), but the difference failed to reach the level of statistical significance (possibly because of the very small count of recurrent cases). The antibody titer was inversely associated with the recurrence rate, but the difference failed to reach the level of statistical significance. Individuals with the lowest immunity (antibody titer in the first tercile) had the highest recurrence rate (1.8%) and the rate declines with increasing immunity to reach its lowest value (0.9%) in those with the highest antibody titer (third tercile). The most notable (though not significant statistically) is the direct association between the duration of risk exposure to reinfection (from the first positive rtPCR test result until the first vaccine dose or the total follow-up for unvaccinated) and recurrence rate. The recurrence rate is lowest (0.1%) among individuals in the first two tercile (smallest duration of risk exposure being < 299 days). This rate jumps to 3.7% when the duration of risk exposure increases beyond 299 days (third tercile) (Table 3).

**Table 3:** The rate of recurrent positive COVID-19 during the unvaccinated period by selected explanatory variables rtPCR: Real-time reverse transcriptase-polymerase chain reaction; NS: Not significant statistically

		Recurrent positive COVID-19 during the unvaccinated period						
		Negative		Positive		Total		
		N	%	N	%	N	%	p
1.	Age group (years)							<0.001
	10-19	193	98.0	4	2.0	197	100	
	20-29	539	98.0	11	2.0	550	100	
	30-39	1405	98.9	15	1.1	1420	100	
	40-49	803	99.0	8	1.0	811	100	
	50+	395	98.8	5	1.3	400	100	
	Total	3335	98.7	43	1.3	3378	100	
2.	Gender							0.16 (NS)
	Female	752	98.7	10	1.3	762	100	
	Male	2583	98.7	33	1.3	2616	100	
3.	Nationality							0.27 (NS)
	Expat	3039	98.8	37	1.2	3076	100	
	Qatari	296	98.0	6	2.0	302	100	
4.	Antibody titer-categories							0.37 (NS)
	First tercile (lowest) <= 19.4	1109	98.2	20	1.8	1129	100	
	Second tercile (average) 19.5-81.04	1108	98.8	13	1.2	1121	100	
	Third tercile (highest) > 81.04	1118	99.1	10	0.9	1128	100	
5.	Duration of risk exposure to reinfection (from the first positive rtPCR test result until the first vaccine dose or the total follow-up for unvaccinated) - categories							0.91 (NS)
	First tercile (lowest) <= 223	1137	99.9	1	0.1	1138	100	
	Second tercile (average) 223-299	1118	99.9	1	0.1	1119	100	
	Third tercile (highest) > 299	1080	96.3	41	3.7	1121	100	

A multivariate model was used to calculate the net and independent effect of each of the previously listed explanatory variables on the risk of experiencing COVID-19 recurrence during a maximum unvaccinated period of one year (Table 4). The model itself was statistically significant and among the five tested explanatory variables, only the longest duration of risk exposure (since the first COVID-19 episode) beyond 299 days (third tercile) had a statistically significant association with recurrence risk. Being in this risk exposure category increases the risk of having recurrent COVID-19 by 51 times compared to those with the shortest risk exposure (first tercile category < 223 days) after adjusting for the possible confounding effect of the remaining explanatory variables included in the model. Compared to the youngest age group (10-19) the oldest group (50+ years) increased the recurrence probability by 2.28 times. Male gender slightly reduced the recurrence risk compared to females (odds ratio = 0.68). Being a Qatari was associated with a 2.3 times increase in the risk of recurrence compared to expats. A higher antibody titer was associated with a marginal reduction in risk when compared to the first tercile category after adjusting for the possible confounding effect of the remaining explanatory variables included in the model.

**Table 4:** The risk of having recurrent positive COVID-19 during the unvaccinated period by selected explanatory variables NS: Not significant statistically; OR: Odds ratio

	Adjusted OR	95% confidence interval OR	p
1. Age group (years)			
(20-29) years compared to the youngest age group (10-19 years)	1.59	(0.47 to 5.39)	0.46 (NS)
(30-39) years compared to the youngest age group (10-19 years)	1.07	(0.33 to 3.48)	0.91 (NS)
(40-49) years compared to the youngest age group (10-19 years)	1.21	(0.34 to 4.29)	0.77 (NS)
(50+) years compared to the youngest age group (10-19 years)	2.28	(0.58 to 9.02)	0.24 (NS)
2. Male gender compared to female	0.68	(0.33 to 1.43)	0.31 (NS)
3. Qatari nationality compared to expats	2.30	(0.9 to 5.91)	0.08 (NS)
4. Antibody titer-categories			
Second tercile (average) 19.5-81.04 compared to the first tercile (lowest) <= 19.4	0.75	(0.37 to 1.54)	0.44 (NS)
Third tercile (highest) > 81.04 compared to the first tercile (lowest) <= 19.4	0.66	(0.3 to 1.47)	0.31 (NS)
5. Duration of risk exposure to reinfection (from the first positive rtPCR test result until the first vaccine dose or the total follow-up for unvaccinated/total follow-up for unvaccinated) - categories			
Second tercile (average) 223-299 compared to first tercile (lowest) <= 223	1.09	(0.07 to 17.53)	0.95 (NS)
Third tercile (highest) > 299 compared to first tercile (lowest) <= 223	50.98	(6.89 to 377.07)	<0.001
Constant	0.001		<0.001

## Discussion

A study by Yang et al. [17] has demonstrated that the antibody response induced by coronaviruses, such as SARS-CoV-2, in humans tends to wane over time and differs between coronavirus types and the severity of the disease. According to a previously published study by Sood et al. [18], all individuals who are either immunized or up to 97% of people who are naturally infected with COVID-19 weeks to months before the antibody tests can have antibodies, including receptor-binding and neutralizing antibodies. These results support the notion that these immune reactions can last for several months. SARS-CoV-2 antibodies, including IgG, can be found in human serum and plasma using the immunoassay known as anti-SARS-CoV-2. The test is meant to assist in determining the immune response to SARS-CoV-2 as mentioned by Gilbert et al. [19]. People who have already had SARS-CoV-2 can be found and the population's exposure to the virus can be estimated using serological tests that look for antibodies against the virus. Understanding the COVID-19 pandemic critically depends on how long adaptive immunity against SARS-CoV-2 lasts and how well it works after the initial infection. Serological antibody tests against SARS-CoV-2 can help identify people who have already had the virus and find out how much of a population has been exposed. As a result, they might influence the choice of whether to apply, enforce, or loosen containment measures. They could thus have an impact on the decision to implement, enforce, or relax containment measures as in the WHO Research and Development Blueprint [20].

The current study, which retrospectively included a sizable cohort of randomly selected individuals, provides important details on the persistence of circulating SARS-CoV-2 antibodies after COVID-19 infection, which wanes over time. In addition, it assessed the risk of reinfection in unvaccinated individuals. Thus, it contributes to providing insight into the seroconversion rate and its protection value in unvaccinated individuals.

The ability of antibodies to bind to antigens gradually grows over time after an infection or vaccination due to a process known as affinity maturation. High-affinity antibodies can lead to neutralization by locating and binding viral epitopes. Like the Payne study, Iwasaki and Yang, and Tay et al. also found that even though correlates of immunity or protection against SARS-CoV-2 have not yet been identified, antibodies are thought to play a significant role in neutralizing the virus [21-23] in SARS-CoV-2 infection, antibodies that target both the spike and nucleocapsid proteins start to form as early as day nine onwards. This correlates with a strong neutralizing response, which suggests seroconversion and may result in protection for at least a brief period. It requires more research in this field to determine whether neutralizing antibodies against SARS-CoV-2 confer long-term immunity [13,24-26].

This study included 3,378 individuals who recovered after COVID-19 infection, out of which the highest numbers were young adults, mostly males between 30 to 49 years old. This was expected since the population was football fans who recovered from COVID-19 and were interested in attending Arab Cup matches [27] as mentioned by Coyle et al. The sensitivity and specificity of the laboratory assays used are necessary to ensure study results' validity. The antibody assay used was among the most effective and scientifically proven commercial platforms with a specificity of > 99.8%. This confirms that false-positive results by cross-reactivity with other common cold coronaviruses are not likely like Jeremijenko et al. [28].

In the current study, the median antibody titer was highest in the older age group > 50 years. A similar conclusion was reported by Coyle et al. [27] which showed that in an urban population of Qatar, men and adults aged 20 to 79 had a higher chance of being seropositive than women and children. Post et al. [1] found that males have a lower risk for recurrent COVID-19 infections as compared to females which may be explained by the finding of higher median antibody titer in males. Qatari nationality was found to have a higher recurrence rate (2%) than non-Qatari expatriates (1.2%). This is possibly explained by finding a significantly higher median antibody titer in the latter. The current study demonstrated that the highest antibody titer was noted during the first tercile and wanes over time. This is in line with other recent studies by Choe et al. [29] about anti-SARS-CoV-2 IgG antibodies targeted against the S protein that have been around for well over a year.

To find any evidence of recurrent infection (a positive rtPCR test result), the health records of the study group were monitored for a maximum of 18 months after serology testing or until the first dose of COVID-19 vaccination. A 1.3% overall recurrence rate was found. The age was significantly associated with the recurrence rate. Younger age groups had a higher recurrence rate. The recurrence rate was inversely proportional to antibody titers in the population which is supported by previous findings by Islam et al. [30] that there is a possibility of reinfection as the humoral immunity weakens over time.

Limitations

Study limitations include a lack of direct neutralization assays and the fact that antibody levels alone do not directly equate to immunity. In addition, the cross-sectional study design, a convenience sample with an unknown degree of selection bias due to public recruitment, self-reported COVID-19 test results, the study population being largely White and healthy, and lack of information on breakthrough infections were other possible biases. Participants were given only one month to complete antibody testing, which may have contributed to the 52% rate among those invited to test.

## Conclusions

The study found a statistically significant association between recurrence risk and the duration of risk exposure since the first COVID-19 episode. Compared to those with the lowest risk of exposure to reinfection (shortest duration after the first infection), those beyond 299 days of at-risk exposure since the first episode have a 51-fold higher risk of developing recurrent COVID-19. In addition, the present study's findings confirm previous research suggesting that immunity developed after primary infection with SARS-CoV-2 may prevent subsequent exposure to the virus at least for a short time. This is in line with previous studies showing that antibody-mediated immunity can provide strong protection against re-infection in seropositive individuals up to nine months post-infection.
